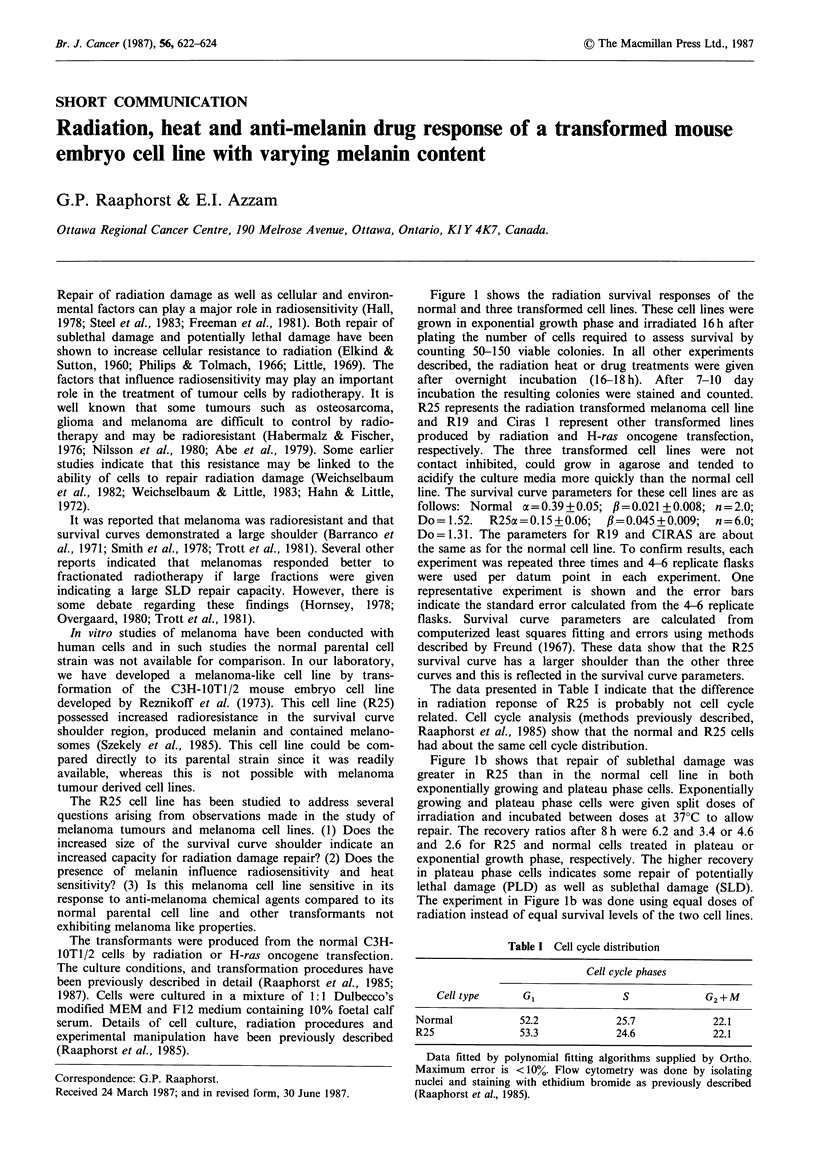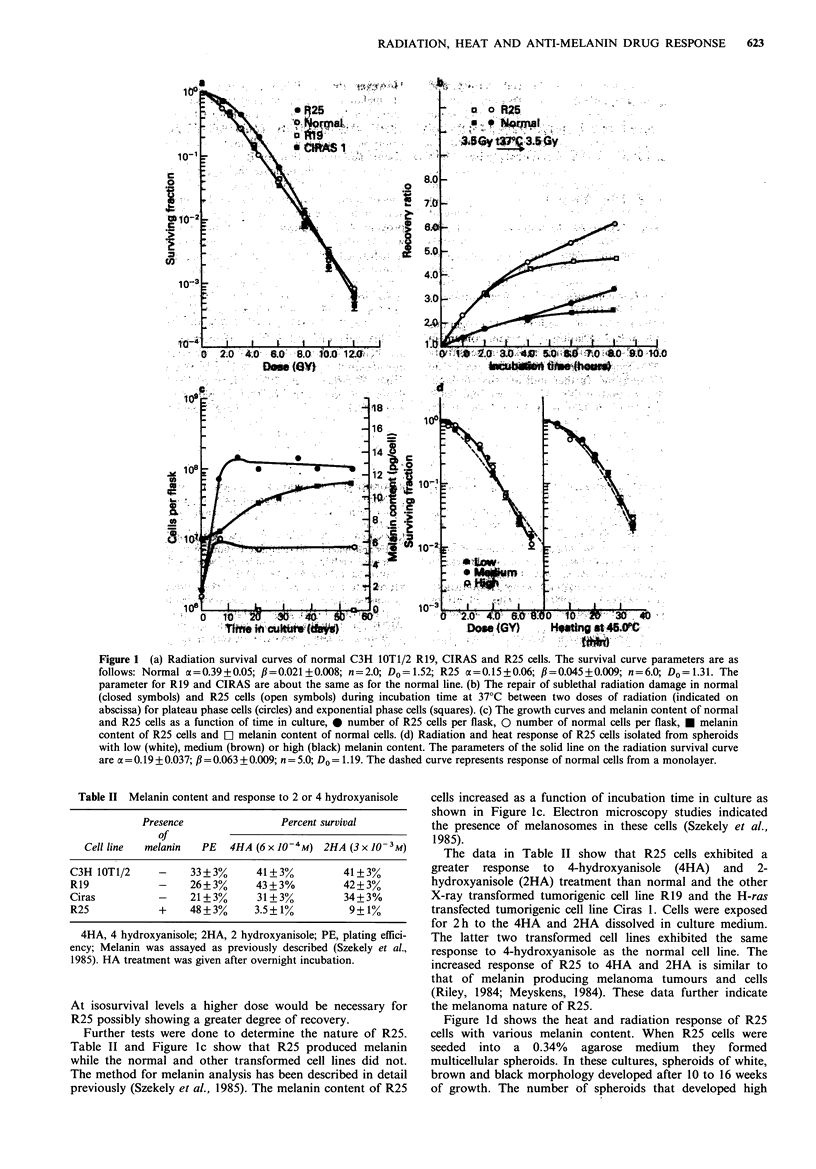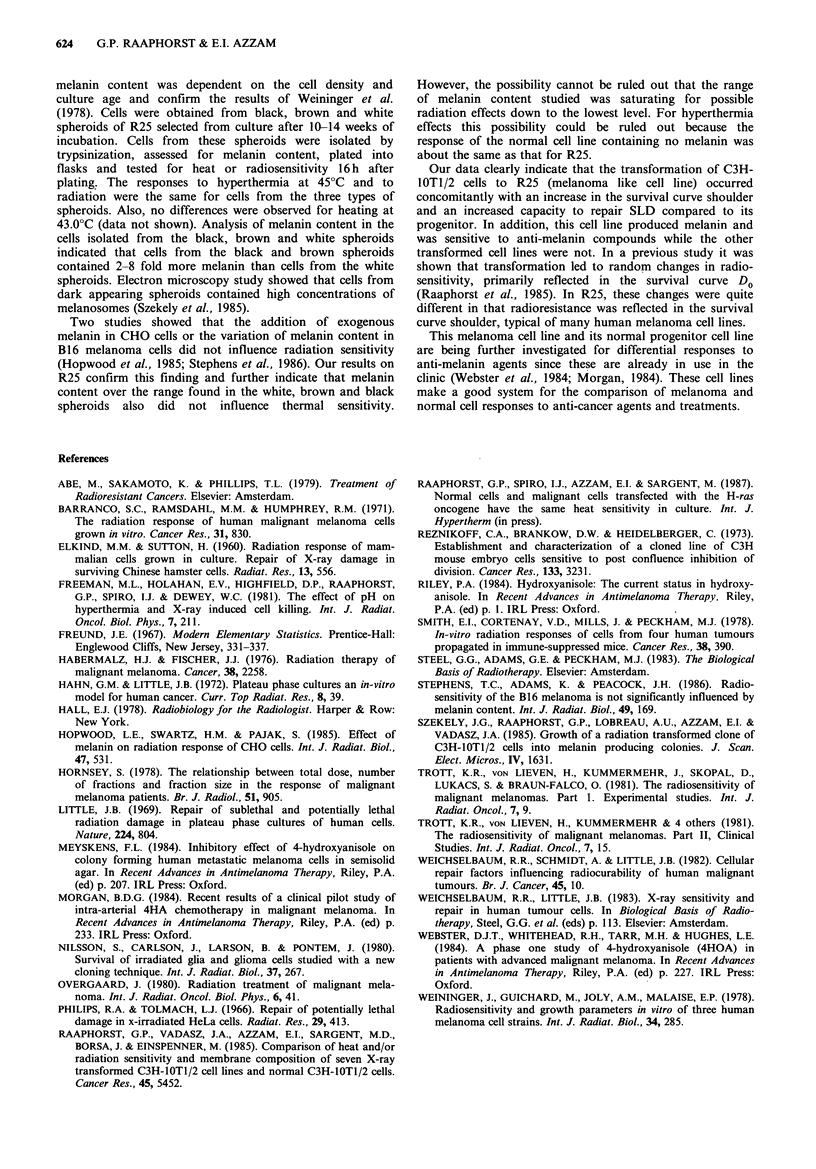# Radiation, heat and anti-melanin drug response of a transformed mouse embryo cell line with varying melanin content.

**DOI:** 10.1038/bjc.1987.254

**Published:** 1987-11

**Authors:** G. P. Raaphorst, E. I. Azzam

**Affiliations:** Ottawa Regional Cancer Center, Ontario, Canada.


*Br. J. Cancer* (1987), **56**, 622–624 © The Macmillan Press Ltd., 1987

SHORT COMMUNICATION

### Radiation, heat and anti-melanin drug response of a transformed mouse embryo cell line with varying melanin content

G.P. Raaphorst & E.I. Azzam

*Ottawa Regional Cancer Centre, 190 Melrose Avenue, Ottawa, Ontario, K1Y 4K7, Canada.*

Repair of radiation damage as well as cellular and environmental factors can play a major role in radiosensitivity (Hall, 1978; Steel *et al.*, 1983; Freeman *et al.*, 1981). Both repair of sublethal damage and potentially lethal damage have been shown to increase cellular resistance to radiation (Elkind & Sutton, 1960; Philips & Tolmach, 1966; Little, 1969). The factors that influence radiosensitivity may play an important role in the treatment of tumour cells by radiotherapy. It is well known that some tumours such as osteosarcoma, glioma and melanoma are difficult to control by radiotherapy and may be radioresistant (Habermalz & Fischer, 1976; Nilsson *et al.*, 1980; Abe *et al.*, 1979). Some earlier studies indicate that this resistance may be linked to the ability of cells to repair radiation damage (Weichselbaum *et al.*, 1982; Weichselbaum & Little, 1983; Hahn & Little, 1972).

It was reported that melanoma was radioresistant and that survival curves demonstrated a large shoulder (Barranco *et al.*, 1971; Smith *et al.*, 1978; Trott *et al.*, 1981). Several other reports indicated that melanomas responded better to fractionated radiotherapy if large fractions were given indicating a large SLD repair capacity. However, there is some debate regarding these findings (Hornsey, 1978; Overgaard, 1980; Trott *et al.*, 1981).

*In vitro* studies of melanoma have been conducted with human cells and in such studies the normal parental cell strain was not available for comparison. In our laboratory, we have developed a melanoma-like cell line by transformation of the C3H-10T1/2 mouse embryo cell line developed by Reznikoff *et al.* (1973). This cell line (R25) possessed increased radioresistance in the survival curve shoulder region, produced melanin and contained melanosomes (Szekely *et al.*, 1985). This cell line could be compared directly to its parental strain since it was readily available, whereas this is not possible with melanoma tumour derived cell lines.

The R25 cell line has been studied to address several questions arising from observations made in the study of melanoma tumours and melanoma cell lines. (1) Does the increased size of the survival curve shoulder indicate an increased capacity for radiation damage repair? (2) Does the presence of melanin influence radiosensitivity and heat sensitivity? (3) Is this melanoma cell line sensitive in its response to anti-melanoma chemical agents compared to its normal parental cell line and other transformants not exhibiting melanoma like properties.

The transformants were produced from the normal C3H-10T1/2 cells by radiation or H-*ras* oncogene transfection. The culture conditions, and transformation procedures have been previously described in detail (Raaphorst *et al.*, 1985; 1987). Cells were cultured in a mixture of 1:1 Dulbecco's modified MEM and F12 medium containing 10% foetal calf serum. Details of cell culture, radiation procedures and experimental manipulation have been previously described (Raaphorst *et al.*, 1985).

Correspondence: G.P. Raaphorst.
Received 24 March 1987; and in revised form, 30 June 1987.

Figure 1 shows the radiation survival responses of the normal and three transformed cell lines. These cell lines were grown in exponential growth phase and irradiated 16 h after plating the number of cells required to assess survival by counting 50–150 viable colonies. In all other experiments described, the radiation heat or drug treatments were given after overnight incubation (16–18 h). After 7–10 day incubation the resulting colonies were stained and counted. R25 represents the radiation transformed melanoma cell line and R19 and Ciras 1 represent other transformed lines produced by radiation and H-*ras* oncogene transfection, respectively. The three transformed cell lines were not contact inhibited, could grow in agarose and tended to acidify the culture media more quickly than the normal cell line. The survival curve parameters for these cell lines are as follows: Normal $\alpha=0.39\pm0.05$; $\beta=0.021\pm0.008$; $n=2.0$; Do = 1.52. R25$\alpha=0.15\pm0.06$; $\beta=0.045\pm0.009$; $n=6.0$; Do = 1.31. The parameters for R19 and CIRAS are about the same as for the normal cell line. To confirm results, each experiment was repeated three times and 4–6 replicate flasks were used per datum point in each experiment. One representative experiment is shown and the error bars indicate the standard error calculated from the 4–6 replicate flasks. Survival curve parameters are calculated from computerized least squares fitting and errors using methods described by Freund (1967). These data show that the R25 survival curve has a larger shoulder than the other three curves and this is reflected in the survival curve parameters.

The data presented in Table I indicate that the difference in radiation reponse of R25 is probably not cell cycle related. Cell cycle analysis (methods previously described, Raaphorst *et al.*, 1985) show that the normal and R25 cells had about the same cell cycle distribution.

Figure 1b shows that repair of sublethal damage was greater in R25 than in the normal cell line in both exponentially growing and plateau phase cells. Exponentially growing and plateau phase cells were given split doses of irradiation and incubated between doses at 37°C to allow repair. The recovery ratios after 8 h were 6.2 and 3.4 or 4.6 and 2.6 for R25 and normal cells treated in plateau or exponential growth phase, respectively. The higher recovery in plateau phase cells indicates some repair of potentially lethal damage (PLD) as well as sublethal damage (SLD). The experiment in Figure 1b was done using equal doses of radiation instead of equal survival levels of the two cell lines.

**Table I** Cell cycle distribution

| *Cell type* | *Cell cycle phases* | | |
|---|---|---|---|
| | $G_1$ | $S$ | $G_2+M$ |
| Normal | 52.2 | 25.7 | 22.1 |
| R25 | 53.3 | 24.6 | 22.1 |

Data fitted by polynomial fitting algorithms supplied by Ortho. Maximum error is <10%. Flow cytometry was done by isolating nuclei and staining with ethidium bromide as previously described (Raaphorst *et al.*, 1985).

**Figure 1** (a) Radiation survival curves of normal C3H 10T1/2 R19, CIRAS and R25 cells. The survival curve parameters are as follows: Normal $\alpha=0.39\pm0.05$; $\beta=0.021\pm0.008$; $n=2.0$; $D_0=1.52$; R25 $\alpha=0.15\pm0.06$; $\beta=0.045\pm0.009$; $n=6.0$; $D_0=1.31$. The parameter for R19 and CIRAS are about the same as for the normal line. (b) The repair of sublethal radiation damage in normal (closed symbols) and R25 cells (open symbols) during incubation time at 37°C between two doses of radiation (indicated on abscissa) for plateau phase cells (circles) and exponential phase cells (squares). (c) The growth curves and melanin content of normal and R25 cells as a function of time in culture, ● number of R25 cells per flask, ○ number of normal cells per flask, ■ melanin content of R25 cells and □ melanin content of normal cells. (d) Radiation and heat response of R25 cells isolated from spheroids with low (white), medium (brown) or high (black) melanin content. The parameters of the solid line on the radiation survival curve are $\alpha=0.19\pm0.037$; $\beta=0.063\pm0.009$; $n=5.0$; $D_0=1.19$. The dashed curve represents response of normal cells from a monolayer.

**Table II** Melanin content and response to 2 or 4 hydroxyanisole

| *Cell line* | *Presence of melanin* | *PE* | *Percent survival* 4HA ($6\times10^{-4}$M) | 2HA ($3\times10^{-3}$M) |
|---|---|---|---|---|
| C3H 10T1/2 | – | 33±3% | 41±3% | 41±3% |
| R19 | – | 26±3% | 43±3% | 42±3% |
| Ciras | – | 21±3% | 31±3% | 34±3% |
| R25 | + | 48±3% | 3.5±1% | 9±1% |

4HA, 4 hydroxyanisole; 2HA, 2 hydroxyanisole; PE, plating efficiency; Melanin was assayed as previously described (Szekely *et al.*, 1985). HA treatment was given after overnight incubation.

At isosurvival levels a higher dose would be necessary for R25 possibly showing a greater degree of recovery.

Further tests were done to determine the nature of R25. Table II and Figure 1c show that R25 produced melanin while the normal and other transformed cell lines did not. The method for melanin analysis has been described in detail previously (Szekely *et al.*, 1985). The melanin content of R25 cells increased as a function of incubation time in culture as shown in Figure 1c. Electron microscopy studies indicated the presence of melanosomes in these cells (Szekely *et al.*, 1985).

The data in Table II show that R25 cells exhibited a greater response to 4-hydroxyanisole (4HA) and 2-hydroxyanisole (2HA) treatment than normal and the other X-ray transformed tumorigenic cell line R19 and the H-*ras* transfected tumorigenic cell line Ciras 1. Cells were exposed for 2 h to the 4HA and 2HA dissolved in culture medium. The latter two transformed cell lines exhibited the same response to 4-hydroxyanisole as the normal cell line. The increased response of R25 to 4HA and 2HA is similar to that of melanin producing melanoma tumours and cells (Riley, 1984; Meyskens, 1984). These data further indicate the melanoma nature of R25.

Figure 1d shows the heat and radiation response of R25 cells with various melanin content. When R25 cells were seeded into a 0.34% agarose medium they formed multicellular spheroids. In these cultures, spheroids of white, brown and black morphology developed after 10 to 16 weeks of growth. The number of spheroids that developed high melanin content was dependent on the cell density and culture age and confirm the results of Weininger *et al.* (1978). Cells were obtained from black, brown and white spheroids of R25 selected from culture after 10–14 weeks of incubation. Cells from these spheroids were isolated by trypsinization, assessed for melanin content, plated into flasks and tested for heat or radiosensitivity 16 h after plating. The responses to hyperthermia at 45°C and to radiation were the same for cells from the three types of spheroids. Also, no differences were observed for heating at 43.0°C (data not shown). Analysis of melanin content in the cells isolated from the black, brown and white spheroids indicated that cells from the black and brown spheroids contained 2–8 fold more melanin than cells from the white spheroids. Electron microscopy study showed that cells from dark appearing spheroids contained high concentrations of melanosomes (Szekely *et al.*, 1985).

Two studies showed that the addition of exogenous melanin in CHO cells or the variation of melanin content in B16 melanoma cells did not influence radiation sensitivity (Hopwood *et al.*, 1985; Stephens *et al.*, 1986). Our results on R25 confirm this finding and further indicate that melanin content over the range found in the white, brown and black spheroids also did not influence thermal sensitivity. However, the possibility cannot be ruled out that the range of melanin content studied was saturating for possible radiation effects down to the lowest level. For hyperthermia effects this possibility could be ruled out because the response of the normal cell line containing no melanin was about the same as that for R25.

Our data clearly indicate that the transformation of C3H-10T1/2 cells to R25 (melanoma like cell line) occurred concomitantly with an increase in the survival curve shoulder and an increased capacity to repair SLD compared to its progenitor. In addition, this cell line produced melanin and was sensitive to anti-melanin compounds while the other transformed cell lines were not. In a previous study it was shown that transformation led to random changes in radiosensitivity, primarily reflected in the survival curve $D_0$ (Raaphorst *et al.*, 1985). In R25, these changes were quite different in that radioresistance was reflected in the survival curve shoulder, typical of many human melanoma cell lines.

This melanoma cell line and its normal progenitor cell line are being further investigated for differential responses to anti-melanin agents since these are already in use in the clinic (Webster *et al.*, 1984; Morgan, 1984). These cell lines make a good system for the comparison of melanoma and normal cell responses to anti-cancer agents and treatments.

### References

ABE, M., SAKAMOTO, K. & PHILLIPS, T.L. (1979). *Treatment of Radioresistant Cancers.* Elsevier: Amsterdam.

BARRANCO, S.C., RAMSDAHL, M.M. & HUMPHREY, R.M. (1971). The radiation response of human malignant melanoma cells grown *in vitro*. *Cancer Res.*, **31**, 830.

ELKIND, M.M. & SUTTON, H. (1960). Radiation response of mammalian cells grown in culture. Repair of X-ray damage in surviving Chinese hamster cells. *Radiat. Res.*, **13**, 556.

FREEMAN, M.L., HOLAHAN, E.V., HIGHFIELD, D.P., RAAPHORST, G.P., SPIRO, I.J. & DEWEY, W.C. (1981). The effect of pH on hyperthermia and X-ray induced cell killing. *Int. J. Radiat. Oncol. Biol. Phys.*, **7**, 211.

FREUND, J.E. (1967). *Modern Elementary Statistics.* Prentice-Hall: Englewood Cliffs, New Jersey, 331–337.

HABERMALZ, H.J. & FISCHER, J.J. (1976). Radiation therapy of malignant melanoma. *Cancer*, **38**, 2258.

HAHN, G.M. & LITTLE, J.B. (1972). Plateau phase cultures an *in-vitro* model for human cancer. *Curr. Top Radiat. Res.*, **8**, 39.

HALL, E.J. (1978). *Radiobiology for the Radiologist.* Harper & Row: New York.

HOPWOOD, L.E., SWARTZ, H.M. & PAJAK, S. (1985). Effect of melanin on radiation response of CHO cells. *Int. J. Radiat. Biol.*, **47**, 531.

HORNSEY, S. (1978). The relationship between total dose, number of fractions and fraction size in the response of malignant melanoma patients. *Br. J. Radiol.*, **51**, 905.

LITTLE, J.B. (1969). Repair of sublethal and potentially lethal radiation damage in plateau phase cultures of human cells. *Nature*, **224**, 804.

MEYSKENS, F.L. (1984). Inhibitory effect of 4-hydroxyanisole on colony forming human metastatic melanoma cells in semisolid agar. In *Recent Advances in Antimelanoma Therapy*, Riley, P.A. (ed) p. 207. IRL Press: Oxford.

MORGAN, B.D.G. (1984). Recent results of a clinical pilot study of intra-arterial 4HA chemotherapy in malignant melanoma. In *Recent Advances in Antimelanoma Therapy*, Riley, P.A. (ed) p. 233. IRL Press: Oxford.

NILSSON, S., CARLSON, J., LARSON, B. & PONTEM, J. (1980). Survival of irradiated glia and glioma cells studied with a new cloning technique. *Int. J. Radiat. Biol.*, **37**, 267.

OVERGAARD, J. (1980). Radiation treatment of malignant melanoma. *Int. J. Radiat. Oncol. Biol. Phys.*, **6**, 41.

PHILIPS, R.A. & TOLMACH, L.J. (1966). Repair of potentially lethal damage in x-irradiated HeLa cells. *Radiat. Res.*, **29**, 413.

RAAPHORST, G.P., VADASZ, J.A., AZZAM, E.I., SARGENT, M.D., BORSA, J. & EINSPENNER, M. (1985). Comparison of heat and/or radiation sensitivity and membrane composition of seven X-ray transformed C3H-10T1/2 cell lines and normal C3H-10T1/2 cells. *Cancer Res.*, **45**, 5452.

RAAPHORST, G.P., SPIRO, I.J., AZZAM, E.I. & SARGENT, M. (1987). Normal cells and malignant cells transfected with the H-*ras* oncogene have the same heat sensitivity in culture. *Int. J. Hypertherm* (in press).

REZNIKOFF, C.A., BRANKOW, D.W. & HEIDELBERGER, C. (1973). Establishment and characterization of a cloned line of C3H mouse embryo cells sensitive to post confluence inhibition of division. *Cancer Res.*, **133**, 3231.

RILEY, P.A. (1984). Hydroxyanisole: The current status in hydroxyanisole. In *Recent Advances in Antimelanoma Therapy*, Riley, P.A. (ed) p. 1. IRL Press: Oxford.

SMITH, E.I., CORTENAY, V.D., MILLS, J. & PECKHAM, M.J. (1978). *In-vitro* radiation responses of cells from four human tumours propagated in immune-suppressed mice. *Cancer Res.*, **38**, 390.

STEEL, G.G., ADAMS, G.E. & PECKHAM, M.J. (1983). *The Biological Basis of Radiotherapy.* Elsevier: Amsterdam.

STEPHENS, T.C., ADAMS, K. & PEACOCK, J.H. (1986). Radiosensitivity of the B16 melanoma is not significantly influenced by melanin content. *Int. J. Radiat. Biol.*, **49**, 169.

SZEKELY, J.G., RAAPHORST, G.P., LOBREAU, A.U., AZZAM, E.I. & VADASZ, J.A. (1985). Growth of a radiation transformed clone of C3H-10T1/2 cells into melanin producing colonies. *J. Scan. Elect. Micros.*, **IV**, 1631.

TROTT, K.R., VON LIEVEN, H., KUMMERMEHR, J., SKOPAL, D., LUKACS, S. & BRAUN-FALCO, O. (1981). The radiosensitivity of malignant melanomas. Part 1. Experimental studies. *Int. J. Radiat. Oncol.*, **7**, 9.

TROTT, K.R., VON LIEVEN, H., KUMMERMEHR & 4 others (1981). The radiosensitivity of malignant melanomas. Part II, Clinical Studies. *Int. J. Radiat. Oncol.*, **7**, 15.

WEICHSELBAUM, R.R., SCHMIDT, A. & LITTLE, J.B. (1982). Cellular repair factors influencing radiocurability of human malignant tumours. *Br. J. Cancer*, **45**, 10.

WEICHSELBAUM, R.R., LITTLE, J.B. (1983). X-ray sensitivity and repair in human tumour cells. In *Biological Basis of Radiotherapy*, Steel, G.G. *et al.* (eds) p. 113. Elsevier: Amsterdam.

WEBSTER, D.J.T., WHITEHEAD, R.H., TARR, M.H. & HUGHES, L.E. (1984). A phase one study of 4-hydroxyanisole (4HOA) in patients with advanced malignant melanoma. In *Recent Advances in Antimelanoma Therapy*, Riley, P.A. (ed) p. 227. IRL Press: Oxford.

WEININGER, J., GUICHARD, M., JOLY, A.M., MALAISE, E.P. (1978). Radiosensitivity and growth parameters *in vitro* of three human melanoma cell strains. *Int. J. Radiat. Biol.*, **34**, 285.